# Integrative Roles of miRNAs and circRNAs in Plant Antiviral Gene Regulation and Autophagy

**DOI:** 10.3390/plants14223541

**Published:** 2025-11-20

**Authors:** Nurgul Iksat, Zhaksat Baikarayev, Oleksiy Shevchenko, Kuralay Zhanassova, Assemgul Bekturova, Sayan Zhangazin, Zhaksylyk Masalimov

**Affiliations:** 1 Rustem Omarov Plant Biotechnology Laboratory, Department of Biotechnology and Microbiology, L.N. Gumilyov Eurasian National University, Astana 010008, Kazakhstan; 2 Department of Virology, ESC «Institute of Biology and Medicine», Taras Shevchenko National University of Kyiv, 01601 Kyiv, Ukraine

**Keywords:** microRNA, circRNA, plant–virus interactions, immunity, autophagy, programmed cell death

## Abstract

Agriculture is still at serious risk from viral infections, particularly in light of climate change and more intensive farming practices. Small non-coding RNAs (sRNAs), in particular microRNAs (miRNAs) and circular RNAs (circRNAs), have emerged as crucial post-transcriptional regulators of plant antiviral defense in this setting. These molecules provide an essential RNA-based immunity layer by regulating hormones, autophagy, redox balance, immunological signaling, and programmed cell death. In this work, we examine the molecular processes through which circRNAs and miRNAs function during viral infection, focusing on how they affect autophagy and systemic acquired resistance (SAR). Through thorough searches of PubMed, Web of Science, and Scopus, we combined findings from peer-reviewed experimental and transcriptomic studies. Our study covers important crops as well as model species (*Arabidopsis thaliana*, *Nicotiana benthamiana*), providing a thorough understanding of sRNA synthesis, target control, and antiviral signaling. By combining previously disparate data, this review provides a coherent framework for understanding how short RNAs affect plant immune responses to viral infections. We highlight key regulatory relationships that need further study and propose that these results can be used as a foundation for new RNA-based biotechnological approaches. By carefully altering RNA regulatory mechanisms, scientists can use this information to help them create more resistant crops.

## 1. Introduction

Viral infections pose a significant threat to global agriculture in the twenty-first century, endangering crop yields and food security. Rapid changes in farming systems and agricultural practices, driven by climate change and increasing human population pressures, have contributed to destructive viral disease outbreaks [1]. Researchers employ different ways of responding to such problems, from genetic modification using chemicals to enzymatic methods. However, one of the most efficient plant defense strategies against viruses is small RNAs. In viral infections, RNA molecules are mRNA templates with essential viral proteins such as movement proteins, coat proteins, and replication enzymes [2]. However, plants have sophisticated defense mechanisms to degrade viral RNA and DNA [3].

Unlike animals, plants lack circulating immune cells, and thus their defense relies on intrinsic molecular surveillance systems capable of distinguishing self from non-self. Viral components are recognized with remarkable precision, initiating a multilayered antiviral immune response. Two major layers of pathogen perception form the basis of this system: pattern-triggered immunity (PTI) and effector-triggered immunity (ETI) [4]. The first layer, PTI, operates through pattern recognition receptors (PRRs), including receptor-like kinases (RLKs) and receptor-like proteins (RLPs), localized in the plasma membrane. These receptors detect conserved viral- or damage-associated molecular patterns (VAMPs/DAMPs), such as double-stranded RNA (dsRNA), coat proteins, or replicative intermediates formed during infection [5]. Activation of PRRs triggers complex intracellular signaling cascades involving mitogen-activated protein kinases (MAPKs), calcium influx, and the generation of reactive oxygen species (ROS), forming the PTI response [6,7,8]. This leads to extensive transcriptional reprogramming, the accumulation of salicylic acid (SA), jasmonic acid (JA), and ethylene (ET), and the induction of defense-related genes that restrict viral replication and movement [9,10].

**Figure 1:**
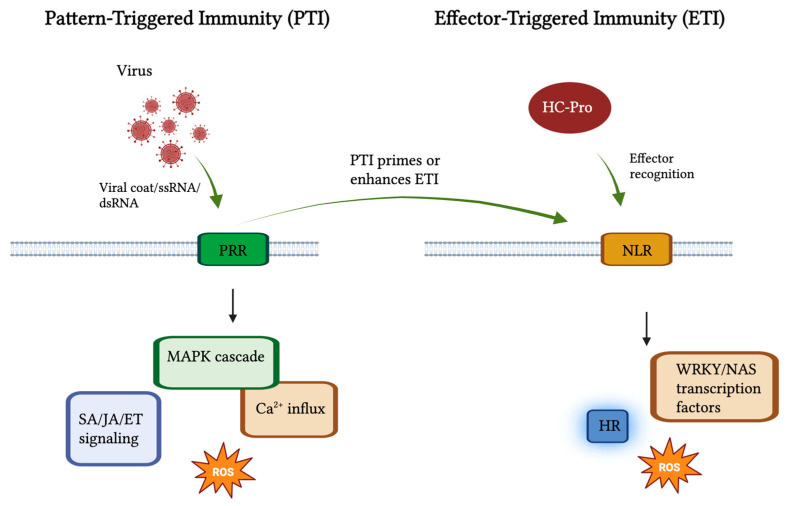
Plant immune response is induced by viral infection. The activation of MAPK cascades, Ca^2+^ influx, reactive oxygen species (ROS), and salicylic acid (SA), jasmonic acid (JA), and ethylene (ET) accumulation is caused by the detection of viral coat proteins or double-stranded RNA/single-stranded RNA by plasma-membrane-bound pattern-recognition receptors (PRRs; RLKs/RLPs) and results in pattern-triggered immunity (PTI). The intracellular nucleotide binding leucine rich repeat (NLR) receptors mediate effector triggered immunity (ETI) which recognize individual viral effectors (HC-Pro) resulting in increased ROS bursts, WRKY and NAC transcription factor activation, and the hypersensitive response (HR). PTI and ETI work together to pay high-quality antiviral defense. Green arrows represent activation; red shapes represent viral components.

The second layer of defense, ETI, is mediated by intracellular nucleotide-binding leucine-rich repeat (NLR) receptors that recognize specific viral effectors—such as movement proteins, silencing suppressors, or replicases—either directly or indirectly via host protein modifications (Figure 1) [11]. Activation of NLRs triggers a strong immune response characterized by a rapid ROS burst, activation of transcription factors (including WRKY and NAC families), and the induction of the hypersensitive response (HR), a localized form of programmed cell death (PCD) that restricts viral spread [11,12,13,14].

In parallel, plants employ RNA silencing, often referred to as a third antiviral layer. Dicer-like (DCL) enzymes process double-stranded viral RNAs into short interfering RNAs (siRNAs), which are incorporated into Argonaute (AGO) proteins to form the RNA-induced silencing complex (RISC) [15]. This complex guides the degradation or translational repression of complementary viral RNAs, constituting a highly specific post-transcriptional defense mechanism [16]. Viruses, however, have co-evolved viral suppressors of RNA silencing (VSRs), such as P19 of tombusviruses or HC-Pro of potyviruses, which inhibit this process by binding sRNAs or blocking AGO activity [17]. Collectively, PTI, ETI, and RNA silencing form an integrated antiviral network that translates viral recognition into transcriptional reprogramming, hormonal signaling, oxidative bursts, and RNA-based silencing [18]. This conceptual framework is essential for understanding the regulatory roles of miRNAs and circRNAs in modulating downstream antiviral responses.

sRNAs in plants represent a critical layer of post-transcriptional regulation, enabling the fine-tuning of physiological processes under biotic and abiotic stress conditions. Their role becomes particularly prominent during viral infections, where sRNAs are central to the orchestration of immune responses, modulation of hormonal signaling pathways, and maintenance of metabolic and energy balance. Viral pathogens, in turn, have evolved diverse strategies to interfere with this regulatory network, highlighting the deep coevolution between viruses and their plant hosts [19,20].

Research has primarily focused on miRNAs, which control the expression of key immune-related regulators, including sensitivity to phytohormones such as salicylic acid, ethylene, and jasmonates; transcription factors from the WRKY, MYB, and NAC families; and antioxidant enzyme cascades. Viral suppressors of RNA silencing often disrupt these pathways by targeting components of the miRNA biogenesis machinery or the RNA-induced silencing complex (RISC), shifting the regulatory balance toward increased susceptibility to infection [21,22].

In parallel, accumulating evidence links miRNA-mediated regulation to redox homeostasis and the activation of programmed cell death (PCD), which serves as a localized containment strategy during viral attack. Reactive oxygen species (ROS)-responsive miRNAs modulate the expression of antioxidant genes, and their dysregulation can trigger cell death in infected tissues. Autophagy is also integrated into this response, functioning in the selective degradation of viral components. However, certain viruses hijack the autophagic machinery to promote their own replication. The selective autophagy receptor NBR1, for instance, mediates the targeting of viral proteins to the vacuole for degradation [23,24].

In recent years, circRNAs have emerged as a distinct and versatile layer of post-transcriptional gene regulation. Characterized by their covalently closed circular structure, circRNAs exhibit exceptional stability and resistance to exonuclease degradation, enabling them to participate in diverse regulatory networks. One of their most prominent molecular functions involves acting as miRNA sponges, sequestering miRNAs and thereby modulating their availability for target mRNA binding and RISC-mediated gene silencing [25]. This competitive interaction adds an additional layer of control to post-transcriptional regulation and highlights the integrative role of circRNAs in fine-tuning gene expression. In plants, circRNAs display tissue-specific and stress-responsive expression patterns, suggesting dynamic regulatory functions under both biotic and abiotic stress conditions. Recent studies have revealed that plant circRNAs are implicated in antiviral defense, potentially modulating host–virus interactions through miRNA-mediated pathways. Due to their high stability and condition-specific expression profiles, circRNAs are increasingly recognized as promising biomarkers for pathogen infection and environmental stress responses [26,27,28].

Taken together, sRNA-mediated regulation constitutes a sophisticated and multilayered system that integrates immune gene networks, hormone signaling, oxidative stress responses, autophagy, and programmed cell death. Elucidating the dynamics of these interactions is fundamental to understanding plant antiviral immunity and holds significant promise for the development of innovative, RNA-based strategies for crop protection.

## 2. Gene Regulation by Small RNAs in Plants

### 2.1. Plant miRNA Biogenesis

Plant miRNAs are sRNA molecules critical for the post-transcriptional regulation of gene expression, primarily involving developmental processes, adaptation to stress conditions, and antiviral defense. MiRNA biogenesis is a complex series of processes that begins in the nucleus and concludes in the cytoplasm, where the mature miRNAs are incorporated into the RISC to down-regulate gene expression [29]. This process is initiated by the transcription of MIR genes by RNA polymerase II into primary miRNA transcripts (pri-miRNAs) that are subsequently capped and polyadenylated [30]. These transcripts are often several hundred nucleotides long and undergo tightly regulated processing steps that ensure precise maturation. The accuracy of this pathway is essential, as misprocessing can lead to the accumulation of aberrant miRNAs or impaired gene regulation [31]. The transcripts possess stem-loop hairpin structures central to their subsequent processing [32].

**Figure 2:**
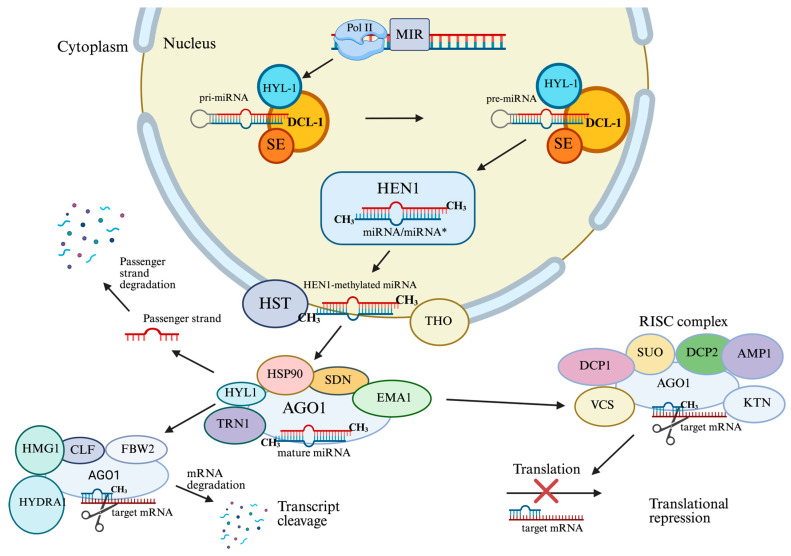
miRNA biogenesis mechanism of plants. DNA-dependent RNA Polymerase II (Pol II) converts miRNAs into primary miRNAs (pri-miRNAs), which are then converted into precursor miRNAs (pre-miRNAs) by DCL-1 complexes. HEN1 methylates the double-stranded miRNA and HST with THO transports the miRNA from the nucleus to the cytoplasm. Then, miRNA would be incorporated into AGO1 thus, forming the RISC. Translational repression and transcript cleavage will take place by the RISC activity. Asterisk (*) indicates the passenger (complementary) strand of the miRNA duplex (miRNA*). “×” symbol represents inhibition of translation in the target mRNA.

Several proteins in the nucleus work together to process pri-miRNAs to pre-miRNAs. These include DICER-LIKE1 (DCL1), an RNase III-type enzyme that cleaves the pri-miRNA; HYPONASTIC LEAVES1 (HYL1), a double-stranded RNA-binding protein that stabilizes the complex; SERRATE (SE), a zinc finger protein that enhances processing accuracy; and TOUGH (TGH), which ensures correct miRNA maturation [33]. As shown in Figure 2, the product ~21-nucleotide miRNA/miRNA * duplex is methylated at 3′ ends by HEN1 to protect it from degradation and uridylation [34]. This methylation event is a distinctive characteristic of plants and is essential to ensure miRNA stability.

Once the miRNA/miRNA duplex is methylated, it is exported to the cytoplasm [35]. Export is facilitated by the plant homolog of exportin-5, namely HASTY (HST), but may involve other proteins [36]. The duplex is then unwound in the cytoplasm [37]. The passenger strand, or miRNA*, is typically degraded, while the guide strand, or the mature miRNA, is incorporated into ARGONAUTE1 (AGO1), the central member of the RISC [38]. The miRNA-containing RISC then selectively silences specific messenger RNAs based on sequence complementarity [39]. The miRNA-mRNA interaction in plants is usually characterized by near-perfect complementarity to enable direct cleavage of the target mRNA. However, in animal systems, the process is often dependent on partial complementarity to gain translational repression instead [40]. This precise binding allows the plant to carefully control the expression of genes that are important for immunity, hormones, and development. MiRNA silencing typically takes place within specialized cytoplasmic domains referred to as processing bodies (P-bodies), which are sites of mRNA degradation or storage [41].

Furthermore, miRNAs in plants could be part of feedback circuits to fine-tune elements of the silencing apparatus itself [42]. Several miRNAs have been shown to target transcripts encoding components of their own processing machinery, including DCL1 and AGO1, establishing feedback regulation that ensures balance within the silencing system [43]. Furthermore, emerging evidence indicates that miRNA biogenesis is dynamically modulated through post-translational modifications of key components such as DCL1, HYL1, SE, and HEN1, allowing plants to adjust miRNA maturation in response to developmental and stress cues [44]. Autoregulatory feedbacks, like these function to balance the miRNA system and fine-tune expression in fluctuating environmental conditions. The highly regulated biogenesis of plant miRNAs ensures that gene regulation is rapid and precise during viral infection, where a swift response is required to contain pathogen spread.

Therefore, a well-regulated miRNA biogenesis serves as the foundation for their functional activity in gene expression regulation, which provides plants with precise control over development and adaptation to external factors.

### 2.2. Functional Roles of microRNAs in Plant Antiviral Immunity

Following their biogenesis, miRNAs enter regulatory cascades that suppress the expression of genes involved in immune responses. During viral infection, these small RNAs function as central mediators of post-transcriptional regulation by modulating hormone signaling pathways [45], redox homeostasis [46], systemic resistance [47], and programmed cell death (PCD) [48], thereby ensuring a coordinated balance between growth and defense.

Among the most extensively studied miRNAs in antiviral defense is miR168, which regulates the expression of AGO1, a core component of the RISC responsible for viral RNA degradation via RNA interference [49]. Upon viral infection, miR168 expression increases, leading to downregulation of AGO1 and attenuation of the immune response [50]. However, plants such as *A. thaliana* finely tune the expression of miR168 and AGO1 to maintain immune homeostasis [51]. Transgenic lines expressing an miR168-resistant AGO1 variant (4m-AGO1) exhibit severe morphogenetic defects, underscoring the essential role of this regulatory axis in both immunity and development [52].

Numerous miRNAs modulate auxin signaling pathways, which are closely linked to plant immunity. For instance, miR393 targets auxin receptors TIR1/AFBs, reducing auxin sensitivity and enhancing resistance to a wide range of pathogens [53]. Simultaneously, miR160 represses ARF10, ARF16, and ARF17, which also contributes to antiviral resistance [54], including protection against tobacco mosaic virus (TMV) [55], soybean mosaic virus (SMV) [56], and mungbean yellow mosaic virus (MYMIV) [57]. During cucumber mosaic virus (CMV) infection, expression levels of both miRNAs shift, indicating their coordinated role in defense [58]. The CMV 2b protein, a well-characterized viral suppressor of RNA silencing (VSR), disrupts miRNA-mediated transcript degradation and inhibits DNA methylation, further destabilizing hormonal and immune balance [59].

miR159 regulates the expression of GAMYB/MYB transcription factors, which are involved in hormonal signaling and stress responses [60]. Among its isoforms, miR159a is most abundantly expressed, and its upregulation is associated with delayed flowering, altered cell cycle progression, and increased susceptibility to PCD, especially during tomato leaf curl virus (ToLCV) infection [61]. Dysregulation of MYB factors impairs hormonal homeostasis and weakens resistance. Similarly, miR172 contributes to both development and immunity by targeting the transcription factor TOE3, which regulates flowering [62]. Overexpression of miR172 facilitates TMV accumulation, while downregulation enhances resistance. These findings highlight the need for strict control of miR172 expression to maintain the growth-immunity balance [63].

miR164 targets NAC transcription factors, which modulate the hypersensitive response (HR) and PCD [64]. Expression of NACs depends on the type of pathogen: for instance, ATAF1 enhances resistance to the biotrophic fungus *Blumeria graminis* [65], but reduces resistance during necrotrophic infection by *Botrytis cinerea* [66], particularly when overexpressed.

miRNAs that regulate oxidative stress play a particularly important role in antiviral defense. In *Oryza sativa*, osa-miR528 suppresses ascorbate oxidase (AO) to maintain ROS homeostasis [67]. Infection with rice tungro bacilliform virus (RTBV) downregulates miR528, resulting in AO accumulation, reduced levels of reduced ascorbate, and a surge in ROS, which initiates PCD [68]. This defense mechanism involves caspase-like protease activity and chromatin condensation to restrict viral spread [69]. Viral proteins further destabilize miR528, exacerbating oxidative imbalance. miR398, another key antioxidant-related miRNA, targets Cu/Zn superoxide dismutases (CSD1 and CSD2) as well as COX5 and BCBP, which are involved in detoxifying ROS [70]. In *A. thaliana*, three isoforms, miR398a, miR398b, and miR398c, regulate these targets differently [71]. Viral infections such as TMV and beet necrosis yellow vein virus (BNYVV) downregulate miR398, leading to ROS accumulation and activation of defense responses [72]. In rice, rice stripe virus (RSV) alters the expression of osa-miR395y, osa-miR167h, and osa-miR7695, disturbing stress gene regulation and triggering PCD [73]. Similarly, in wheat, reduced expression of miR1119 increases MYC2 levels and enhances the activity of antioxidant enzymes (CAT, POD, SOD), boosting stress resistance [74].

miRNAs also contribute to SAR [56]. For example, miR166 regulates HD-ZIP III transcription factors, which are involved in xylem differentiation and shoot apical meristem development [75]. The AGO10 protein binds miR166 to promote HD-ZIP III expression and maintain meristem integrity. If miR166 is loaded into AGO1 instead, or if AGO10 is inactivated, systemic signaling and meristem maintenance are impaired [76]. Such disruptions may occur during tomato bushy stunt virus (TBSV) infection, allowing the virus to manipulate host architecture [77]. miR319, which regulates development via TCP transcription factors, also plays a role in immunity [78]. During TuMV and GBNV infections, systemic repression of miR319 results in TCP accumulation and cell death [79]. In *Vigna unguiculata*, reduced expression of miR319a.2 activates metacaspase-8, which initiates PCD a process similar to what has been observed in *Arabidopsis* under abiotic stress [80].

Viruses actively manipulate host miRNA expression. Some encode viral miRNAs that target plant defense genes. For instance, *Geminiviridae* viruses (ACMV and EACMV-UG) produce miRNAs that repress host transcription factors and signaling proteins. In response, plants activate conserved miRNAs such as miR159, miR156, and miR171, which target viral RNAs [81]. Viral suppressors like TBSV P19 bind 21-nt miRNA/siRNA duplexes, preventing AGO loading and silencing. This suppression disrupts cellular homeostasis, enhances viral replication, and promotes tissue necrosis. Additional miRNAs, such as miR162 and miR845, modulate RNAi and genome stability [82]. miR162 regulates DCL1, a key enzyme in miRNA processing [83]. Viral suppression of miR162 impairs the generation of virus-specific siRNAs, reducing RNAi efficacy. miR845 targets LTR retrotransposons, and its activity may be influenced by circular RNAs, linking antiviral defense to genome integrity and cell fate [84].

In Chinese cabbage infected with TuMV, high-throughput sequencing revealed 86 conserved and 45 novel miRNAs, 69 of which were differentially expressed. Their predicted targets include genes involved in development, stress response, and defense. Notably, some of these miRNAs are responsive to both viral and cold stresses, indicating crosstalk between different regulatory pathways [85]. Similar expression patterns occur in ToLCV-infected plants, where upregulation of miR159 and downregulation of miR164 and miR171 affect transcription factors (MYB, AP2, SBP) and genes related to morphogenesis and oxidative stress [86].

**Figure 3:**
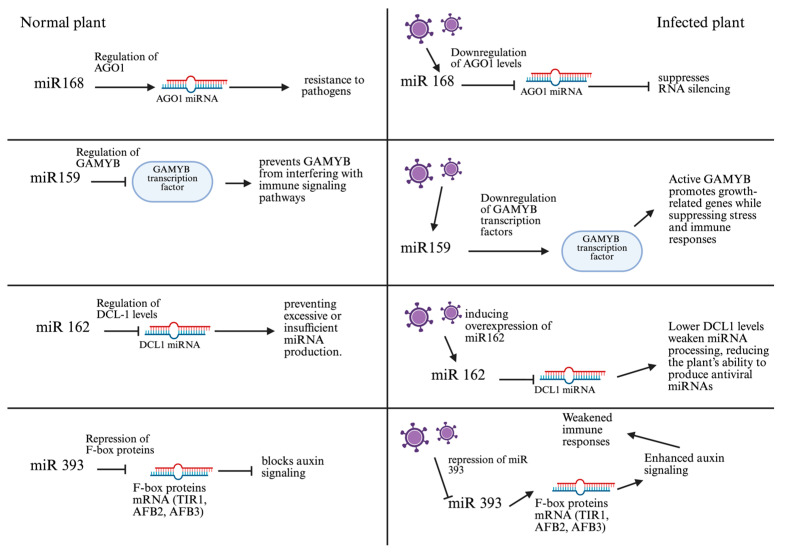
A comparative model illustrating how miR168, miR159, miR162, and miR393 regulate antiviral defense in healthy and virus-infected plants. Under normal conditions, these miRNAs maintain immune balance by controlling AGO1, GAMYB, DCL1, and F-box proteins. During viral infection, altered expression disrupts RNA silencing, enhances growth and auxin signaling, and weakens immune responses. Purple virus icons represent infection, arrows (→) indicate activation, and blunt-ended lines (⊣) denote inhibition.

Together, these findings demonstrate that miRNAs form a multilayered regulatory network orchestrating immune responses during viral infection (Figure 3). By integrating hormonal pathways, redox signaling, development, and systemic immunity, miRNAs enable efficient defense while minimizing trade-offs with growth.

Viruses, in turn, have evolved diverse strategies to disrupt, hijack, or subvert miRNA-mediated regulation (Table 1). A deeper understanding of these mechanisms offers promising opportunities for engineering virus-resistant crop varieties. Moreover, emerging evidence suggests that miRNAs closely interact with other classes of non-coding RNAs, including circRNAs, which further contribute to antiviral regulation and introduce an additional layer of complexity in host–virus interactions.

### 2.3. Roles of Plant Circular RNAs in Regulating Programmed Cell Death During Viral Infection

**Figure 4:**
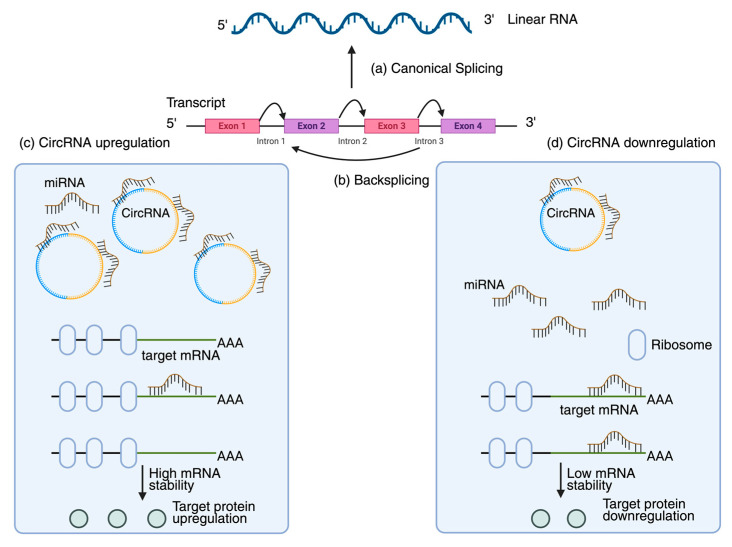
Biogenesis and regulatory roles of circRNAs. (**a**) Canonical splicing produces linear RNAs, while (**b**) backsplicing forms covalently closed circRNAs. (**c**) Upregulated circRNAs sponge miRNAs, enhancing target mRNA stability and protein production. (**d**) Downregulation releases miRNAs, increasing mRNA degradation and reducing protein synthesis. Exons are shown as colored boxes, circular arrows represent backsplicing events, and blue and orange arcs mark exon junctions.

CircRNAs, formed from the backsplicing of pre-mRNA, are covalently closed RNA molecules devoid of a 5′ cap and a 3′ poly(A) tail. They have recently been identified in numerous plants and associated with various physiological processes, including antiviral defense and stress responses [87,88]. CircRNAs are potential regulators of plant immune responses owing to their tissue-specific, stress-induced expression and remarkable resistance to degradation [89]. CircRNAs are essential in regulating gene transcription, particularly for immune response-related genes, and in competitive endogenous RNA networks by functioning as «sponges» for miRNAs (Figure 4) [90]. CircRNA is crucial to post-transcriptional regulation through the activity of decoy miRNAs, especially in contexts of stress response. Specialized databases and tools such as PlantCircNet [91] and GreenCircRNA [92] have been created to visualize regulatory networks and forecast circRNA roles as miRNA sponges, facilitating a comprehensive analysis of circRNA interactions with miRNAs and mRNAs. The researchers identified 83 circRNAs in TYLCV samples utilizing the CircRNA Identifier (CIRI) tool [93]. Current research on viral circRNAs suggests that they may emulate host circRNAs to evade immune responses, inhibit RNA silencing, or act as decoys to sequester host miRNAs. In contrast to animals, this domain remains inadequately comprehended in plants; yet, substantial evidence indicates that circRNA contributes to biotic defense. Rice infected with the brown planthopper insect vector exhibited varied expression of 186 circRNAs, some of which regulated pathways associated with immune response [94]. Besides PlantCircNet and GreenCircRNA, there are many databases that store data related to plant RNA viruses, as well as the interactions between hosts and viral RNAs. For example, Q-bank Plant Viruses and Viroids provide information about plant viruses and viroids, including species-specific data, DNA barcodes, and sequence records [95]. Likewise, the PAmiRDB (Plant Antiviral miRNA Database) is a compilation of genomic and proteomic annotations of plant RNA viruses [96]. DPVweb is a major source of information on viruses, viroids, and viral satellites in plants, fungi, and protozoa [97]. Together, these resources complement circRNA databases, providing a broad computational base to study the mechanisms of antiviral RNA actions (Table 2).

**Figure 5:**
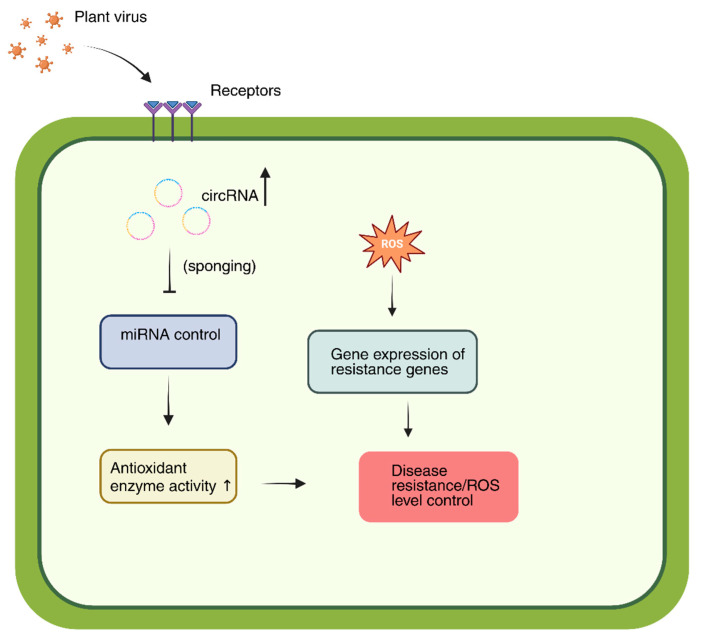
Model of circRNA-mediated antiviral regulation in plants. Upon viral infection, circRNAs are upregulated and act as molecular sponges for specific miRNAs. This reduces miRNA repression of key target genes, including antioxidant enzymes and resistance-related genes. As a result, ROS levels are modulated, defense gene expression is activated, and antiviral resistance is enhanced. Upward arrows indicate the upregulation of circular RNAs and antioxidant enzyme activity in response to viral infection.

Furthermore, circRNAs are considered potential biomarkers of viral infection. They may be utilized for early diagnosis or to assess plant resistance to viruses owing to their stability and specific expression in response to infections [98]. This idea is corroborated by experimental data: rice contains a circRNA from the WRKY9 locus that encodes the 88-amino acid peptide WRKY9 88aa. The production of this circRNA enhanced resistance to several infections, including the rice stripe mosaic virus (RSMV), by augmenting the immune response and generating higher levels of reactive oxygen species [99]. Viral infections can modify the circRNA expression profile in plants at the population level. The quantity of circRNAs identified in *Zea mays* infected with the maize Iranian mosaic virus (MIMV) increased to 1443 from 1165 in the control group [100]. Comparable results were observed in the *A. thaliana* model, where sequencing identified several circRNAs with differential expression across various viral infections [101]. Furthermore, investigations into plant vectors confirmed their involvement in the infectious process. Specifically, circRNA2030 expression increased in the midgut of RBSDV-infected *Laodelphax striatellus*, and its silencing via RNA interference intensified the infection and reduced the expression of the parent gene, phospholipid transfer ATPase (PTA). MiRNAs that interacted with circRNA2030 did not show an increase in expression after its suppression, indicating an indirect regulatory influence [102]. CircRNAs are a promising category of regulatory RNAs in plants that modulate immune gene expression and engage in endogenous RNA networks to facilitate antiviral defense (Figure 5) [103,104,105]. However, additional comprehensive research is required to completely elucidate the mechanisms governing their direct interaction with plant viruses.

Against this background, the effect of miRNA on autophagy induced by plant viruses is of particular interest, which opens a new direction for studying the integration of RNA regulation and antiviral defense mechanisms.

### 2.4. Complex Interaction of Viruses and Autophagy

**Figure 6:**
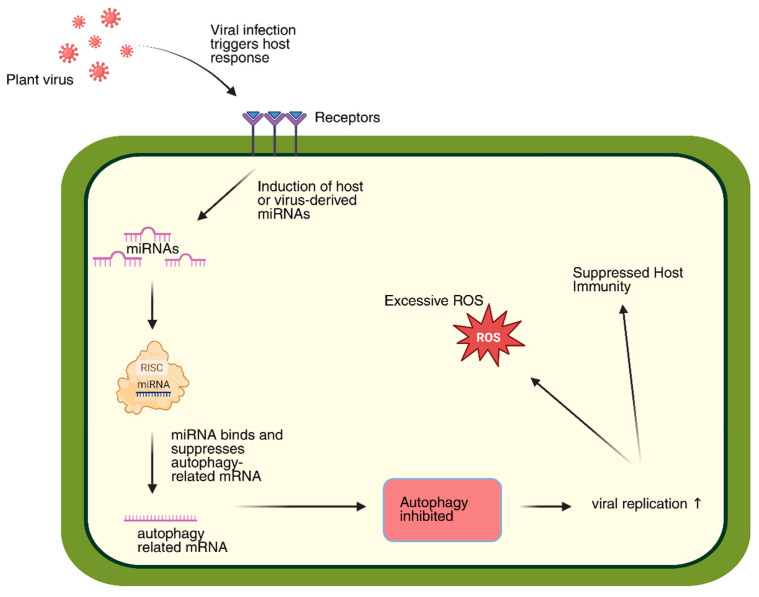
Schematic representation of plant viral infection-induced suppression of autophagy via miRNA regulation. Viral infection triggers host responses leading to induction of host or virus-derived miRNAs, which suppress autophagy-related mRNAs through RISC-mediated binding. This results in inhibited autophagy, excessive ROS accumulation, suppressed host immunity, and enhanced viral replication. Upward arrows indicate increased viral replication.

Autophagy is a highly conserved catabolic mechanism that degrades deleterious protein aggregates, damaged or superfluous organelles, and invading pathogens within lysosomes or vacuoles [106]. This process is essential for maintaining cellular homeostasis and ensuring the quality control of proteins and RNAs. In both plants and animals, autophagy contributes to antiviral defense by removing viral particles, proteins, and replication intermediates (Figure 6).

Initiation of autophagy is orchestrated by the ATG1/ULK1 kinase complex together with other autophagy-related (ATG) proteins, which recruit damaged organelles or viral components to the phagophore assembly site (PAS) [106]. While autophagy possesses inherent antiviral activity, numerous viruses have evolved strategies to circumvent or exploit this pathway to promote infection [107]. Some viral pathogens hijack autophagic processes to facilitate their own replication [108]. A well-characterized example of this duality is provided by the interaction between A. thaliana and turnip mosaic virus (TuMV). The viral 6K2 protein triggers the unfolded protein response (UPR), leading to the transcriptional activation of NBR1, a selective autophagy receptor. NBR1 simultaneously interacts with ATG8f and the viral replicase NIb, thereby promoting the assembly of replication complexes at the tonoplast membrane and accelerating viral replication [109]. This illustrates the ambivalent nature of autophagy in plant–virus interactions: while it can restrict viral accumulation, it may also be subverted to support viral propagation.

In plants, autophagy contributes to antiviral defense through the degradation of essential viral components, including RNA silencing suppressors, movement proteins, and replicases [110,111]. Nevertheless, several viruses have developed mechanisms to counteract this process. For instance, the TuMV VPg protein promotes the autophagic degradation of SGS3, a key factor in RNA silencing [112]. Other viral effectors directly block autophagy: the C4 protein of cotton leaf curl Multan virus (CLCuMuV) interacts with eIF4A to suppress autophagic activity [113], whereas the γb protein of barley stripe mosaic virus (BSMV) disrupts the ATG7-ATG8 interaction, preventing autophagosome formation [114,115]. Similarly, tomato spotted wilt virus (TSWV) induces ATG gene expression but its NSs protein antagonizes this effect by suppressing the ATG6-dependent signaling cascade [116]. In insect vectors, autophagy can also be co-opted, rice gall dwarf virus (RGDV) induces autophagy in host insect cells, thereby facilitating viral dissemination [117]. Recent studies suggest the existence of virus-specific selective autophagy pathways. For example, the plant autophagy receptor NbP3IP mediates degradation of the p3 protein of rice stripe virus (RSV), thereby restricting infection [118]. However, viruses frequently evolve countermeasures to bypass such host defenses [119]. The interaction of tombusviruses with the autophagic machinery further highlights the concept of “neo-phagy” whereby viruses exploit autophagy without being degraded [120,121]. Beyond protein interactions, miRNAs add another regulatory layer to autophagy during viral infection. TuMV, for example, generates virus-derived miRNAs (v-miR-S1 and v-miR-S2) that target host stress-response genes such as HVA22D, potentially modulating autophagic activity and attenuating host immunity [122]. These findings support the notion that plant miRNAs play a central role in post-transcriptional regulation of antiviral responses and influence the crosstalk between autophagy and viral infection.

The interplay between autophagy, miRNAs, and plant viral infection is increasingly well defined but remains incompletely understood. Future research will require integrative approaches, such as CRISPR/Cas-mediated genome editing, degradome sequencing, and AGO immunoprecipitation to identify miRNA targets within autophagy pathways and experimentally validate their roles in plant antiviral defense.

## 3. Conclusions

**Figure 7:**
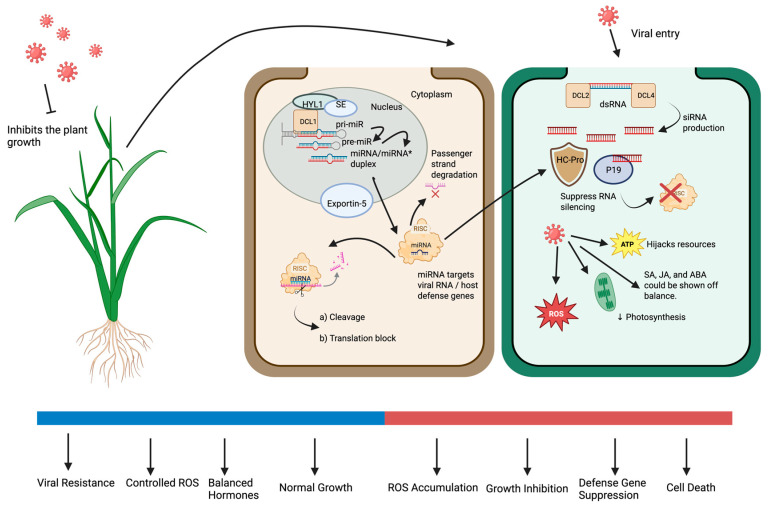
The mechanisms through which plants use microRNAs to respond to viral infection and the mechanisms used by viruses to subvert them. In the nucleus, microRNAs are transcribed and processed into mature forms on the left and then loaded into the RNA-induced silencing complex (RISC). These microRNAs direct RISC to viral RNAs or host defense genes, causing transcript splicing or translational inhibition, which leads to antiviral resistance and the maintenance of hormonal and reactive oxygen species (ROS) homeostasis. Viruses, on the right, enter plant cells and form their own double-stranded RNA (dsRNA), inducing the activation of small interfering RNAs (siRNAs). However, they also generate RNA silencing viral suppressors, including HC-Pro and P19, that block the RISC-mediated mechanism and promote infection. This interference disrupts hormonal signaling such as salicylic acid, jasmonic acid, and abscisic acid increases ROS accumulation, decreases photosynthetic performance, and suppresses genes associated with defense. The final outcome depends on the equilibrium between successful microRNA-mediated reactions and viral suppression, resulting either in viral resistance, where the plant continues normal growth, or in stress responses, growth inhibition, and cell death. The downward arrow indicates reduced photosynthesis, and the ‘×’ symbol denotes inhibition of the RISC complex. Asterisk (*) in miRNA/miRNA* refers to the passenger (complementary) strand of the miRNA duplex that is typically degraded, while the guide strand (miRNA) is incorporated into the RISC complex.

Small RNAs (sRNAs), particularly microRNAs (miRNAs) and circular RNAs (circRNAs), form an interconnected regulatory network that coordinates plant antiviral responses. These molecules act through mutual and complementary regulatory mechanisms, integrating multiple layers of defense, including immune signaling, hormonal homeostasis, autophagy, and programmed cell death (PCD) (Figure 7). CircRNAs function as endogenous modulators of miRNAs, sequestering specific miRNAs and thereby adjusting their accessibility to target messenger RNAs. This fine-tuning of gene expression contributes to stress adaptation and antiviral immunity. In turn, miRNAs regulate genes involved in circRNA biogenesis and stability, creating feedback regulatory loops that dynamically coordinate plant responses during viral infection. Such cross-regulation maintains a balance between energy metabolism, reactive oxygen species (ROS) homeostasis, and defense gene activation, preventing excessive cell death while sustaining efficient antiviral resistance.

Recent advances in CRISPR/Cas-based genome editing and artificial microRNA (amiRNA) design provide new opportunities to manipulate these integrative sRNA networks for improved crop resilience. For instance, suppression of miR168 using a MIM168 construct enhanced rice resistance to *Magnaporthe oryzae*, accelerated flowering, and increased yield through modulation of the AGO1 pathway [123]. Likewise, changes in miR162 expression, which regulates DCL1, have been observed during viral infection and represent additional targets for precise genetic control [124]. Moreover, CRISPR/Cas13a and CasRx systems have been successfully applied to degrade viral RNAs in plants without affecting endogenous transcripts [125,126].

In parallel, artificially engineered miRNAs (amiRNAs) have emerged as an effective and highly specific approach for conferring virus resistance in crops. Transgenic *Nicotiana benthamiana* plants expressing amiRNAs targeting Cucumber mosaic virus or Watermelon silver mottle virus displayed strong and sequence-specific resistance [127,128]. Similarly, polycistronic amiRNA constructs introduced into barley and wheat conferred broad-spectrum resistance to Wheat dwarf virus and Wheat streak mosaic virus, maintaining stability even under low-temperature conditions [129,130]. The design of such amiRNA constructs has been supported by computational platforms such as AmiRNA Designer, WMD3, and AMIRdesigner, which facilitate in silico selection of thermodynamically stable and low off-target sequences [131,132].

Overall, miRNAs and circRNAs act as interdependent regulators that integrate transcriptional and post-transcriptional layers of defense during viral stress. Their interaction connects viral recognition with the regulation of autophagy, hormone signaling, and cell-fate control, ensuring a balanced and efficient immune response. Continued investigation of these interconnected mechanisms, together with the development of CRISPR/Cas and RNA-based delivery technologies (such as RNAi sprays), will support the creation of next-generation virus-resistant crops adapted to the challenges of climate change and global food security.

## Figures and Tables

**Table 1:** Roles of various miRNAs in plant defense.

miRNA	Targets	Related Mechanism Against Viruses	Viruses
miR168	AGO1	Maintains proper AGO1 levels to ensure stable antiviral RNA silencing activity and efficient degradation of viral RNAs	TBSV, TNV, CMV (Tombusvirus, Tobamovirus, Cucumovirus)
miR159	R2R3 and MYB transcription factors	Regulates MYB transcription factors involved in gibberellin and abscisic acid (ABA) signaling pathways, thereby suppressing viral gene expression and limiting replication	CMV, TYLCV (Cucumovirus, Geminivirus)
miR393	TIR1, AFB2, AFB3	Suppresses expression of F-box proteins (TIR1, AFB2, AFB3), blocking the auxin signaling pathway and triggering systemic acquired resistance (SAR) through SA-dependent signal transduction	CMV, TuMV (Cucumovirus, Potyvirus)
miR166	Class III Homeodomain- Leucine Zipper (HD-ZIP III) transcription factors	Regulates HD-ZIP III transcription factors associated with hormonal and vascular development; modulation of their expression enhances SA-dependent SAR signaling and promotes PR-gene expression under viral infection	CMV, BCTV (Cucumovirus, Curtovirus)
miR160	ARF10, ARF16, ARF17	Modulates ARF10/16/17 to balance auxin-mediated growth and SA-mediated defense responses, thereby enhancing immune signaling and restricting viral replication	TuMV, CMV (Potyvirus, Cucumovirus)
miR398	CSD1, CSD2, CSD3	Regulates antioxidant enzymes CSD1 and CSD2 to control reactive oxygen species (ROS) levels and alleviate virus-induced oxidative stress, stabilizing plant defense responses	CMV, TMV (Cucumovirus, Tobamoirus)
miR172	APETALA2-like transcription factors	Suppresses APETALA2-like transcription factors, modulating ethylene-response factor (ERF) signaling to reduce viral load and enhance plant tolerance	TuMV, CMV (Potyvirus, Cucumovirus)
miR162	DCL1	Regulates DCL1 expression to prevent overaccumulation and maintain balanced RNA-silencing activity against viral RNA genomes	CMV. TMV (Cucumovirus, Tobamoirus)

**Table 2:** Online Databases for Plant Virus–miRNA Interaction and Functional Analysis.

Database	Focus	Main Features	Example Use
PlantCircNet (http://bis.zju.edu.cn/plantcircnet/index.php (accessed on 5 November 2025))	ciRNA-miRNA-mRNA network visualization	Visualization of circRNA-miRNA regulatory interactions	Identify miRNA sponges in plants
Q-bank Plant Viruses & Viroids (https://qbank.eppo.int/ (accessed on 6 November 2025))	Prioritizes viruses and viroids that are subject to plant health regulations	Provides taxonomic, biological, and regulatory information on plant viruses and viroids, including curated DNA barcodes and sequence data	Obtain DNA barcodes and validated protocols for rapid and accurate identification
PAmiRDB (https://bioinfo.icgeb.res.in/pamirdb/index.html (accessed on 10 November 2025))	miRNAs and their predicted targets in virus genomes	Contains over 2600 plant miRNAs and their predicted targets across approximately 500 viral species	Identify plant miRNAs predicted to target the virus’s genes
DPVweb (http://www.dpvweb.net/ (accessed on 10 November 2025))	Gives a curated information on complete or nearly complete sequences of plant, fungal, and protozoan viruses, viroids, and satellites, currently covering around 9000 entries	Each entry includes start and end positions of genes and non-coding regions, checked for accuracy, with standardized gene/protein nomenclature within genera and families	Retrieve all annotated gene and protein sequences for that virus

## Data Availability

No new data were created or analyzed in this study. Data sharing is not applicable to this article.

## Abbreviations

The following abbreviations are used in this manuscript:

sRNA	small RNA
miR	microRNA
circRNA	circular RNA
SAR	systemic acquired resistance
RISC	RNA-induced silencing complex
PCD	programmed cell death
ROS	reactive oxygen species
VSR	viral suppressor of RNA silencing
HR	hypersensitive response
